# Nutritional Support in Malnourished Children With Compromised Gastrointestinal Function: Utility of Peptide-Based Enteral Therapy

**DOI:** 10.3389/fped.2021.610275

**Published:** 2021-06-07

**Authors:** Mukadder Ayse Selimoglu, Aydan Kansu, Sema Aydogdu, Aysugul Alptekin Sarioglu, Simge Erdogan, Buket Dalgic, Aysel Yuce, Fugen Cullu Cokugras

**Affiliations:** ^1^Department of Pediatric Gastroenterology, Atasehir and Bahcelievler Memorial Hospitals, Istanbul, Turkey; ^2^Department of Pediatric Gastroenterology, Ankara University School of Medicine, Ankara, Turkey; ^3^Department of Pediatric Gastroenterology, Ege University Faculty of Medicine, Izmir, Turkey; ^4^Abbott Laboratories, Istanbul, Turkey; ^5^Department of Pediatric Gastroenterology, Gazi University Faculty of Medicine, Ankara, Turkey; ^6^Department of Pediatric Gastroenterology, Hacettepe University Faculty of Medicine, Ankara, Turkey; ^7^Department of Pediatric Gastroenterology, Istanbul University Cerrahpasa Faculty of Medicine, Istanbul, Turkey

**Keywords:** malnutrition, gastrointestinal function, children, nutritional support, malabsorption, diarrhea, peptide-based enteral therapy

## Abstract

This review focuses on nutritional support in malnourished children with compromised gastrointestinal function addressing the interplay between malnutrition and gastrointestinal dysfunction, and the specific role of peptide-based enteral therapy in pediatric malnutrition. Malnutrition is associated with impaired gut functions such as increased intestinal permeability, malabsorption, and diarrhea, while pre-existing functional gastrointestinal disorders may also lead to malnutrition. Presence of compromised gastrointestinal function in malnourished children is critical given that alterations such as malabsorption and increased intestinal permeability directly interfere with efficacy of nutritional support and recovery from malnutrition. Appropriate nutritional intervention is the key step in the management of malnutrition, while alterations in gastrointestinal functions in malnourished children are likely even in those with mild degree malnutrition. Therefore, nutritional therapy in children with compromised gastrointestinal function is considered to involve gut-protective interventions that address the overlapping and interacting effects of diarrhea, enteropathy and malnutrition to improve child survival and developmental potential in the long-term. Peptide-based enteral formulas seem to have clinical applications in malnourished children with compromised gastrointestinal function, given their association with improved gastrointestinal tolerance and absorption, better nitrogen retention/ balance, reduced diarrhea and bacterial translocation, enhanced fat absorption, and maintained/restored gut integrity as compared with free amino acid or whole-protein formulas.

## Introduction

Malnutrition remains a major public health problem and a significant cause of mortality among children under five in low- and middle-income countries (LMICs), accounting for 45% of child deaths globally (1–4).

Given the failure of global interventions to improve nutritional status and linear growth, factors that drive and sustain malnutrition have increasingly been addressed in epidemiologic and pathogenesis research (5). Accordingly, the role of adequate nutrition for preserving gastrointestinal function, malnutrition-related gastrointestinal alterations as well as the role of preexisting gastrointestinal dysfunction in development of malnutrition have become increasingly recognized (5, 6).

The gut mucosa serves as a semipermeable barrier permitting nutrient absorption and regulating immune surveillance, while retaining potentially harmful microbes and environmental antigens within the intestinal lumen by preventing their translocation across the epithelial barrier (7, 8). The intestinal barrier function is regulated by multidirectional interactions between epithelial cells, the enteric nervous and the immune system (7, 8).

Indeed, growth and nutritional status impairment is also possible in children with functional gastrointestinal disorders, emphasizing that gastrointestinal dysfunctional alterations may appear as a consequence or cause of the malnutrition (9).

On one hand, malnutrition causes alterations in gastrointestinal digestive and absorptive functions [i.e., reduced pancreatic exocrine function, villous atrophy, increased intestinal permeability, loss of digestive enzymes, malabsorption, and diarrhea; (6, 10)]. Thus, malnutrition in pediatric or adult age leads to severe intestinal mucosal abnormalities along with malabsorption of carbohydrate, fat, protein and other nutrients such as vitamins (6, 10).

On the other hand, environmental enteric dysfunction (EED) with findings suggestive of impaired gut function (i.e., alterations in intestinal structure, function, and immune activation and poor growth) is considered as an important contributor to childhood malnutrition and stunting across geographically widespread resource-limited settings (5, 11–13). In fact, impaired absorption of available nutrients provided during treatment and refeeding-mediated diarrhea risk are considered likely to affect the recovery from malnutrition. Consideration of intestinal functional abnormalities is therefore considered important in selection of the most appropriate diet in treating the acutely ill patient (3). Hence, given that compromised gastrointestinal function interferes with efficacy of nutritional support and recovery from malnutrition (14), development of novel strategies and further interventions are needed to introduce gut-protective therapies targeting inflammation, malabsorption and microbial translocation to reduce morbidity and mortality from diarrhea, enteropathy, and malnutrition (3, 15).

This review will focus on nutritional support in malnourished children with compromised gastrointestinal function, addressing bidirectional interplay between malnutrition and gastrointestinal dysfunction, and the specific role of peptide-based enteral therapy in pediatric malnutrition.

## Bidirectional Interplay Between Malnutrition And Gastrointestional Functions

### Malnutrition-Related Alterations in Gastrointestinal Function

Gastrointestinal aspects of malnutrition refer to operation of adaptation mechanisms to protein-calorie deficiencies and the environment, as well as to the direct relationship between the degree of nutritional deficiency and the severity of gastrointestinal dysfunction (16).

**Figure 1:**
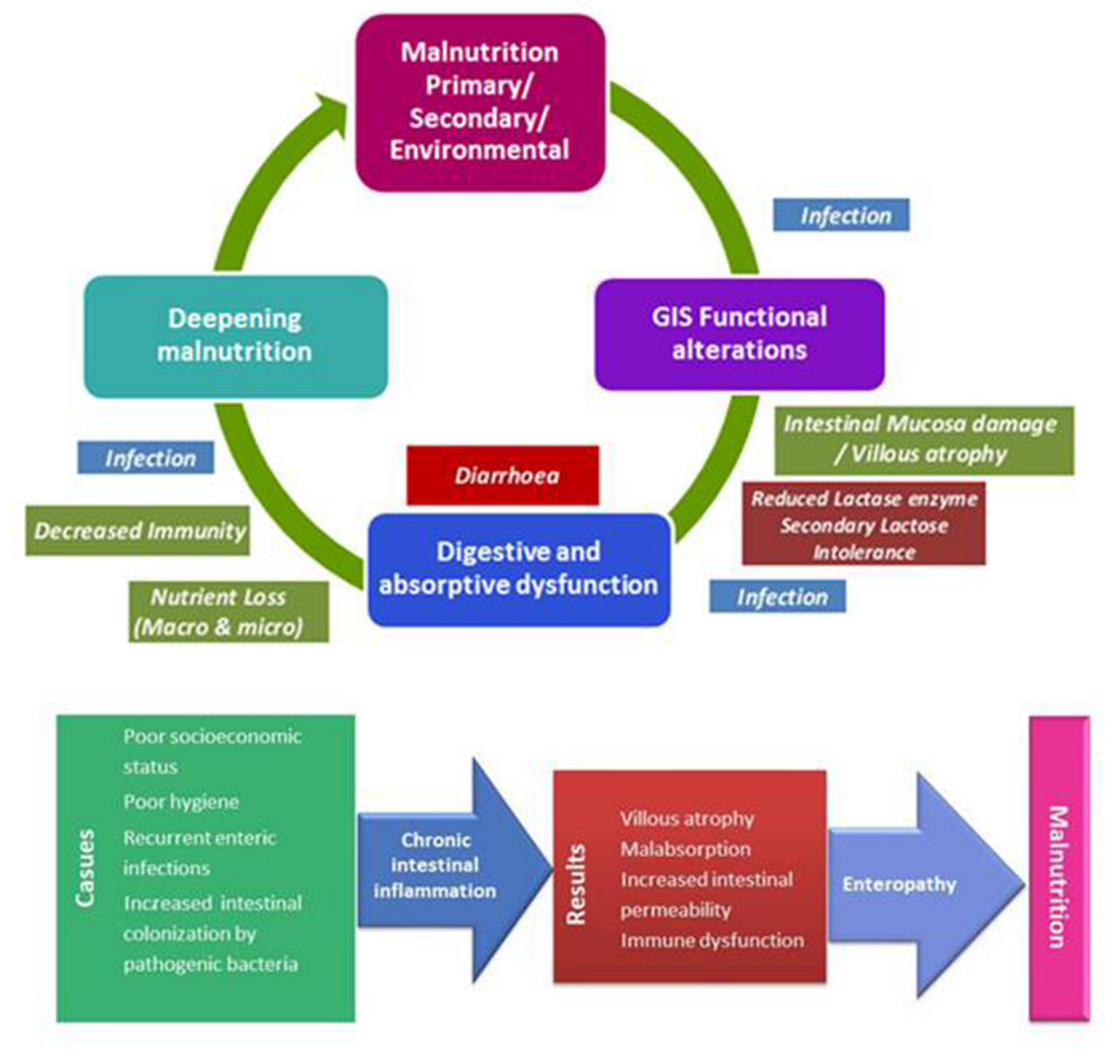
Interplay between malnutrition and gastrointestinal functional alterations; environmental enteric dysfunction.

Malnutrition has been documented to be associated with pancreatic exocrine insufficiency, altered intestinal blood flow, villous atrophy, and increased intestinal permeability which eventually lead to loss of digestive enzymes, secondary lactose intolerance, loss of colonic absorptive function, and diarrhea (6, 10). In addition, malnutrition is suggested to alter protective host factors by causing hypochlorhydria, altered gut motility, reduced antibody synthesis and impaired cell immunity and thereby favoring intestinal colonization by the pathogens (17). Hence, diarrhea is frequently observed and associated with very high burdens of intestinal infection and a high mortality rate in severely malnourished patients [(6, 10, 15, 18); Figure 1].

#### Digestive Functions

The alterations in digestive physiology in childhood malnutrition is considered to be a combination of two synergistic factors including (1) malnutrition-dependent impaired reabsorption of bile salts, excessive bile salt deconjugation, pancreatic exocrine insufficiency (reduced lipase, trypsin, chymotrypsin, and amylase secretion) and impaired intestinal cell function (reduced disaccharidase content, terminal ileal dysfunction) and (2) intestinal bacterial overgrowth and frequent bouts of diarrhea resulting in impaired gut defense and thus increasing the likelihood of further alterations of the microbiota as well as the diarrhea (15, 16).

#### Absorptive Functions

Malnutrition is associated with thinning of the entire intestinal wall and the mucosal lining along with reduced height of the brush border and predominance of cuboidal rather than columnar mucosal cells (16). Permeability of the intestinal mucosa is considered to be 3 times greater in severely malnourished non-critically ill pediatric patients (19), while increased gut permeability is suggested to affect the absorption and metabolism of amino acids, proteins, carbohydrates, lipids, and other nutrients (20, 21).

Correlations between several villus morphometry parameters and measures of gut function were reported in severely malnourished children, including positive correlations between villus height (VH) and villus perimeter (VP; epithelial surface area), between permeability marker lactulose/rhamnose ratio (L/R) and gut microbial translocation indicator lipopolysaccharide binding protein (LBP), between LBP and villus width (inflammation) as well as negative correlations between the L/R and VP and between LR and VH (22).

##### Carbohydrate Absorption

Malnutrition is associated with small intestinal villous blunting that leads to reduced intestinal capacity for monosaccharide and disaccharide absorption (23). Consequent malabsorption of carbohydrate is suggested to contribute to osmotic diarrhea due to water retention induced by accumulation of unabsorbed carbohydrate within the bowel and thus an accelerated flow of fluid down the intestine (14, 15, 23). It has also been suggested that overgrowth of bacteria within the upper small intestine may also lead to malabsorption of carbohydrate and diarrhea (14). In malnourished children with severe protein deficiency, a marked decrease in D-xylose and glucose absorption has also been reported indicating a severe derangement in mucosal cell function, alongside a faster recovery of facilitated diffusion of D-xylose before the recovery of active transport of glucose (16, 24).

##### Protein Absorption

In malnourished children, protein malabsorption is considered likely due to pancreatic insufficiency and small intestine villus atrophy as well as increased protein loss due to increased intestinal permeability (21). Protein deficiency results in deficiency of specific essential amino acids, aggravating gastrointestinal mucosal atrophy (16, 18).

In fact, any impairment in protein digestion and absorption is considered more likely in severely malnourished children and in case of concomitant diarrhea, as associated with increased total fecal nitrogen, increased protein loss from the gut or to a minor impairment in protein digestion and/or amino acid absorption (16). Excessive loss of albumin resulting in both diarrhea and hypoalbuminemia is considered to suggest the presence of a protein losing enteropathy (25), while a direct correlation between the fecal weight in 24 h and the total amount of fecal nitrogen and alpha-1 antitrypsin level is noted in the presence of diarrhea (16).

##### Fat Absorption

**Figure 2:**
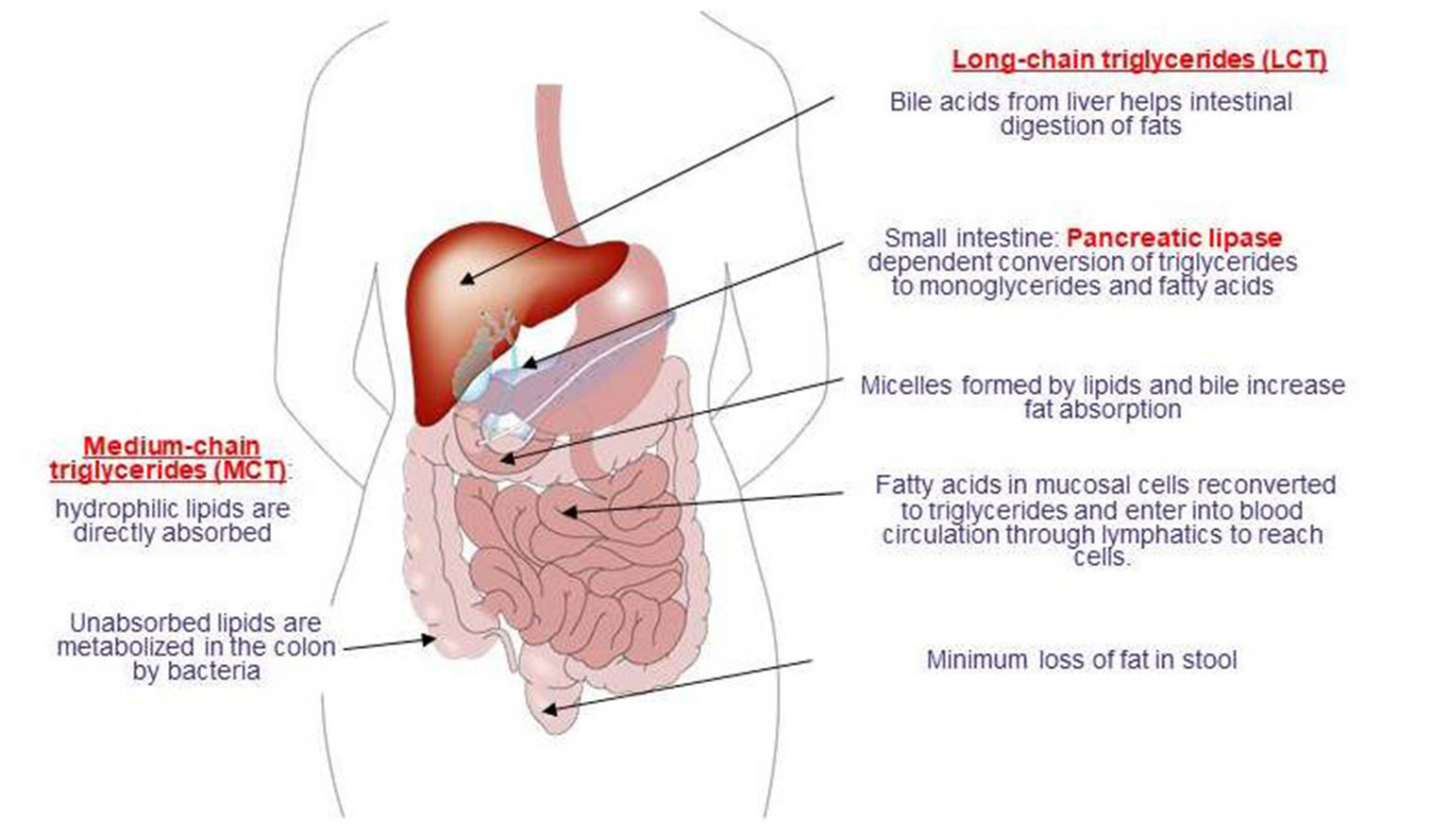
Digestion and absorption of fats.

Fat absorption is markedly impaired in over 50% of children with severe malnutrition (24, 26). Abnormalities in the gastrointestinal handling of lipid along with impaired solubilization or hydrolysis are considered two factors that contribute to malabsorption of fats [(26); Figure 2].

Notably, the severity of steatorrhea relates directly to the degree of protein deficiency and affects triglycerides and free fatty acids, emphasizing that lipolytic activity is not the primary cause of the increased fecal fat in malnutrition (16). Bacterial overgrowth in the small bowel in children with severe malnutrition is also considered likely to contribute directly to ineffective solubilization, digestion, and absorption of lipids (26–28). In addition, diarrhea related reduction in the concentration of conjugated bile acids is also considered to cause deconjugation of the bile salts required for fat absorption that leads to steatorrhea which is a well-recognized accompaniment of malnutrition (14, 16). Vitamin absorption also accompanies malnutrition, while malabsorption of the fat-soluble vitamins A, D, and K is considered likely to be related to steatorrhea (14).

Notably, improvement in fat absorption is considered to occur concomitantly with protein repletion, reaching normality in the absence of diarrhea and after restoration of body protein (16, 24).

### Malnutrition as a Result of Gastrointestinal Dysfunction—EED

Preexisting gastrointestinal dysfunction itself may also lead to an increase in morbidity and mortality secondary to malnutrition, infection, and multi-organ dysfunction if not recognized and treated accordingly (29).

EED is used to describe group of findings suggestive of impaired gut function (i.e., alterations in intestinal structure, function, and immune activation and poor growth) in many children across geographically widespread resource-limited settings (5). EED, a subclinical chronic condition characterized by inflammation of the small bowel mucosa, villous atrophy, dysfunctional nutrient absorption, and increased intestinal permeability, is an emerging contributor to early childhood malnutrition and childhood stunting in resource-limited settings [(5, 20, 21, 30, 31); Figure 1].

Chronic fecal-oral transmission of pathogens is considered the principal factor underlying EED (20, 32). EED is associated with increased gut permeability and alterations in the absorption and metabolism of amino acids, proteins, lipids, carbohydrates, and other nutrients with consequent changes in important metabolites acting in growth and differentiation and gut function and integrity (20). The strong association of EED with childhood stunting (20, 21, 32) seems notable given that stunting is considered to be the commonest presentation of malnutrition that occurs via complex interactions between genetic and environmental factors, recurrent infections and poor nutrition (3).

Although malabsorption and chronic inflammation arising from microbial translocation across an impaired gut barrier is suggested as the causal pathway for the link between EED and stunting (3, 32, 33), interventions aiming to reduce gut permeability and improving linear growth among children in low-income countries have generally failed to achieve both targets (33). Thus, the exact biological mechanism underlying the impact of EED on linear growth remains unknown (3, 32, 33).

## Nutritional Support In Malnourished Children With Compromised Gastrointestinal Function

The adverse impact of metabolic stress and illness on the gut integrity increase the risk of malnutrition via loss of appetite, increased intestinal permeability, diarrhea, and malabsorption of nutrients, whereas those at risk for malnutrition are already at risk for compromised gut function including mucosal intestinal abnormalities and malabsorption of nutrients and vitamins (3, 34).

Accordingly, bidirectional interplay between malnutrition and compromised gastrointestinal function emphasizes the importance of appropriate nutritional support in malnourished children that addresses the overlapping and interacting effects of diarrhea, enteropathy, and malnutrition to improve child survival and developmental potential in the long-term (3, 25, 35).

### Standard Enteral Formulas and Elemental Diets Consisting Solely of Free Amino Acids

Most commercially available enteral formulas consist of whole protein, carbohydrates, and fat (36). Specially formulated ready-to-use therapeutic food (RUTF) including F-75 (75 Kcal energy and 5% protein) and F-100 (100 Kcal energy and 12% protein) are commonly used in outpatient treatment of malnourished children a “starter” feeding formula during initial stage of recovery and as a as a “catch-up” formula in the rehabilitation phase, respectively (37–39).

Formulas consisting of protein in the form of free amino acids (FAA) are also available for patients with malabsorption, food allergy, or specific organ diseases [i.e., renal or hepatic failure; (36)]. These elemental diets aim to provide predigested protein to patients with impaired mucosal absorption (36, 40). However, utilization of elemental diets consisting solely of free amino acids have poor taste and might induce vomiting, diarrhea, and electrolyte abnormalities due to their high osmolality, which limits much of their potential benefits (36, 40). In fact, the specific and discrete uptake systems in GI tract are based on easier and uniform absorption of small peptides consisting of 4–12 amino acids compared to corresponding mixtures of FAAs, while bacterial translocation was reported to be 5 times more likely in animals fed FAA-based vs. peptide-based diets (41–43).

Due to presence of impaired gut function with severe mucosal abnormalities and malabsorption in malnourished children, standard enteral formulas may not be well-tolerated (34). Dilution of formulas to achieve adequate tolerance and to prevent diarrhea is not an appropriate approach in malnourished children given that they are already nutritionally compromised and this dilution results in lower levels of nitrogen intake and prolonged negative nitrogen balance (34, 36).

In a past study with 400 children (aged 12–59 months) living in rural Malawi, authors reported EED in 80.7% of children and identified 77 metabolites that were either negatively (dietary polyphenols, citrulline, ornithine, tryptophan, and indolelactate) or positively (acylcarnitines, intermediates of β-oxidation of fatty acids, deoxycarnitine, trimethylamine-N-oxide, metabolites from ω-oxidation of fatty acids, odd-chain fatty acids, cystathionine, and homocitrulline) associated with gut permeability (44). The authors suggested that EED is a syndrome characterized by secondary carnitine deficiency, alterations in polyphenol and amino acid metabolites, abnormal fatty acid oxidation and metabolic dysregulation of sulfur amino acids, tryptophan, and the urea cycle (44).

Notably, supplementation with glutamine, tryptophan, and leucine has been suggested to ameliorate the mucosal pathology by increasing villus height in adults with EED and to improve barrier function when combined with micronutrient supplementation (45). Hence amino-acid based supplementation is considered to affect villus morphology without necessarily affecting permeability, mucosal or systemic inflammation, malabsorption, and intestinal microbiota (45, 46), while villous atrophy has also been suggested to have a protective role in EED (47). Accordingly, availability of nutrients such as glutamine, leucine, and tryptophan is suggested to be a rate-limiting impact on villous height (45).

Some studies suggested that glutamine can reduce intestinal permeability in malnourished children and those with EED (48, 49), while negative data are also evident indicated that glutamine supplementation failed to improve growth or intestinal status in malnourished Gambian infants (50). In addition, alanyl-glutamine supplementation was reported to improve permeability and child weight but does not promote linear growth, and this limited benefit is suggested to be due to ongoing damage from intestinal inflammation (32, 51).

Notably, oral replenishment of specific amino acids (such as alanyl-glutamine) is considered likely to support intestinal epithelial cell proliferation and restoration of host growth, depending on combining treatment with targeted antibiotics and/or modulators against host inflammation (5, 52, 53).

Experimental studies indicated the association of parenteral delivery of arginine with decreased intensity of Cryptosporidium infection and the severity of growth impairment in undernourished mice through induction of defense-promoting nitric oxide pathway and the arginase pathway that is important for intestinal epithelial cell restitution (5, 54). Tryptophan was reported to increase systemic and mucosal T-regulatory cell counts in piglets, regardless of the nutritional status (55), whereas tryptophan has also been considered likely to aggravate certain infections such as Cryptosporidium during protein malnutrition (5, 56).

### Peptide-Based Enteral Therapy

Peptide-based formulas contain proteins that have been hydrolyzed to produce peptides of varying lengths (34). Presence of protein in the form of small peptides (dipeptides and tripeptides) in peptide-based formulas seems to be advantageous, since absorption of small peptides is considered to occur more rapidly and efficiently and to be less severely affected by pathologic states as compared with free amino acid absorption (34, 36, 41).

Indeed, non-competitive specific peptide carrier systems that are independent of free amino acid carriers have been identified in the intestinal brush border, and their importance has been confirmed in patients with impaired intestinal absorption (36).

Providing protein in a form of a protein hydrolysate is suggested to directly affect nitrogen utilization and retention, regardless of the protein quantity or the non-protein calorie-to nitrogen ratio of a particular enteral formula (36). Clinical trials have documented association of a peptide-based diet with 16 times greater nitrogen retention and decreased ureagenesis than a free amino acid diet (57), with a significantly enhanced rate of absorption and a decrease in albumin clearance during luminal perfusion than a standard polymeric diet (37, 58) and with a significant reduction in stool output than a standard enteral alimentation (59).

Accordingly, the development of peptide-based enteral formulas is considered a significant milestone in the advancement of clinical nutrition, being associated with improved outcomes as compared with free amino acid or whole-protein formulas in terms of improved gastrointestinal tolerance (60–63), nitrogen retention/ balance (34, 36), visceral protein synthesis and absorption (38), lower risk of diarrhea and bacterial translocation (34, 36, 61, 62), and better maintained/restored gut integrity (34, 60–62). These findings suggest that the enhanced absorption of protein in the form of a protein hydrolysate and its effect on ureagenesis may have clinical applications in patients with compromised gastrointestinal functions such as impaired digestion or absorption and those with a protein losing enteropathy (36, 63). Indeed, failure to thrive (transitional feeding) is considered amongst the indications for use of a peptide based formula (36, 63), and the benefit of enteral therapy with peptide-based formulas was also reported in children with compromised gastrointestinal function (35).

This seems important given that protein hydrolysates are also considered to improve sodium and water absorption alongside nutrient absorption depending on the type of protein substrate used for the production of hydrolysate (i.e., casein and lactalbumin hydrolysates), while protein hydrolysates with a higher concentration of small peptides are also associated with a greater stimulatory effect on nutrient as well as on sodium and water absorption (36). Given that enteral formula intolerance was frequently manifested as diarrhea, the effect of peptides on jejunal water absorption is considered likely to be beneficial (36).

The carbohydrate component of peptide-based formulas is quite similar (range, 127–189 g/L), mainly consisting of glucose oligosaccharides, which are easily metabolized and broken down during luminal hydrolysis and mucosal absorption (36).

The quantity of fat provided in the peptide-based formulas ranges from 10 g/L fat up to over 50 g/L fat. Although it is still controversial whether the quantity of fat affects absorption, use of medium chain triglycerides (MCT) as a fat source in all peptide-based diets (up to 40–70%) is considered very important given the likelihood of MCT to further enhance lipid absorption, particularly in patients with underlying malabsorption syndromes (36, 64, 65).

The smaller molecular weight of MCTs than long-chain triglycerides (LCTs) facilitates the action of pancreatic lipase and thus enables MCTs to be hydrolyzed both faster and more completely than LCTs with a reduction in stool lipid excretion (26, 66). Notably, a 40% contribution of MCT to fat intake was reported to enhance fat absorption by about 10% relative to formulae based on LCTs (67), while the absorption of calcium, magnesium and amino acids has also been reported to be enhanced when the diet contains MCTs, particularly in infants (66, 68).

Although an increase in the energy density of foods and thus provision of adequate energy in diet is often achieved by increasing the lipid content (26), in children with severe malnutrition, who are most in need of additional dietary energy, there is disturbed lipid metabolism (26). MCT component of peptide-based formulas is important in this regard, given that MCTs are considered a preferable source of abundant and rapidly available energy in case of increased energy needs [i.e., undernourished patients after major surgery or children during normal or retarded growth; (66, 68, 69)].

Use of peptide-based formula vs. standard whole (intact) protein formula has been associated with reduced rate of tube feeding diarrhea (44.0 vs. 0.0%) in ICU patients (70), while superiority of dipeptide- and tripeptide-based enteral formulas over whole-protein formulas in terms of efficacy and tolerability was also reported in malnourished abdominal surgery patients (60).

Peptide-based formulas, as compared with FAA or whole-protein formulas, were reported to improve nitrogen balance and visceral protein synthesis, to reduce diarrhea and bacterial translocation, to maintain or restore gut integrity and to improve outcomes (71).

Immune-enhancing formulas contain specific immune modulating nutrients (i.e., arginine, glutamine, omega 3 fatty acids, and/or dietary nucleotides), while the protein composition can vary [i.e., whole proteins plus FAA or a combination of whole proteins, peptides and FAA; (42)]. Data from studies that combine dietary supplementation with additional protein or other complementary foods revealed an increase in childhood growth (72–75), in contrast to limited or clinically insignificant role of micronutrients (72, 76). A daily supplement of bovine colostrum with egg powder (BC/egg) for 3 months in infants with EED was reported to be associated with in less linear growth faltering as compared to controls (77). Given the association of deficiencies in the tryptophan-kynurenine-niacin pathway with EED, use of immunomodulatory micronutrients (i.e., nicotinamide) in treating or preventing EED is currently under investigation (78).

Although the exact pathophysiological mechanisms remain unknown, the switch from a catabolic to an anabolic state via rapid refeeding in a starved patient is considered likely to cause of the clinical manifestations of the refeeding syndrome (RFS) (79, 80). While the carbohydrates play the key role in RFS via stimulation of insulin secretion and thus emergence of a sudden shift from fat to carbohydrates metabolism, proteins may also affect the insulin secretion (79, 80). Nonetheless, while the isocaloric properties of peptide-based formulas seems to offer a steady increase in calorie intake, currently there is no evidence regarding the potential advantages or disadvantages of using peptide-based formula in the RFS.

In fact, in addition to favorable tolerability, digestion, and absorption characteristics, peptide-based diets have also been reported to be superior over AAF, parenteral nutrition and regular oral diets in terms of weight gain and growth, reduction of the systemic inflammatory response and lower mortality rates across a multitude of patient populations (61). Accordingly, while peptide formulas, are more expensive (approximately five-fold) than whole-protein formulas, they are considered to shorten the average ICU stay by about 1 day with likely impact on total cost and quality of care (60). Although no cost-effectiveness studies are available, peptide based formulas may enable cost savings through improved gastrointestinal tolerance, lower morbidity, and faster recovery.

### Enteral Nutrition and Intestinal Dysbiosis

A number of reports in pediatric patients with Crohn's disease indicated the likelihood of enteric nutrition to result in significant and relevant alterations in the intestinal microbiota, such as reduction in bacterial diversity and a loss of stability (81). Enteral nutrition has also been suggested to alter the microbiome among pediatric Crohn's disease patients resulting in an unexpected decrease in beneficial bacterial taxa (*Faecalibacterium prausnitzii* and Bacteroides), despite clinical improvement, particularly in those proceeded to have a subsequent sustained remission (82–85).

The effect of nutritional supplementations on microbiota has also been reported in experimental studies. For example, the dietary emulsifiers (i.e., carboxymethylcellulose, gum Arabic, soy lecithin/polysaccharide, glycerol derivatives) added to enteral formulations to extend their shelflife and texture has been associated with intestinal dysbiosis by reducing the alpha diversity and microbial stability of the intestinal microflora, promoting colitis, and metabolic syndrome (86, 87). Moreover, in a study with preterm infants, authors reported that phase-specific changes in the microbiota were significantly associated with the ratio of lipids (Actinobacteria and Proteobacteria), proteins (Firmicutes), or carbohydrates (Actinobacteria, Proteobacteria, and Firmicutes) in enteral nutrition, along with increase (with greater amounts of lipid) and decrease (with greater amounts of protein) in the abundance of Bifidobacterium, which is an Actinobacterium related to development and maintenance of the healthy infant gut microbiota (88). In fact, given that enteral nutrition contains relatively few components than a regular diet, a reduced alpha diversity of the gut microbiota is considered to be an expected finding under this condition (89). Nonetheless, the exact role of enteric nutrition in the interaction between the mucosal changes and the microbiota remains unknown, necessitating further studies investigating these complex interactions (81).

## Conclusion

Malnutrition is associated with gastrointestinal alterations such as increased intestinal permeability, malabsorption and diarrhea (6, 10). EED is a of impaired gut function and an important contributor to childhood malnutrition and stunting (5, 11–13), which seems particularly important given the refractoriness of stunting to nutrition-specific interventions (10). Presence of compromised gastrointestinal function in malnourished children is critical given that alterations such as malabsorption and increased intestinal permeability directly interfere with efficacy of nutritional support and recovery from malnutrition (14). Nutritional therapy in children with compromised gastrointestinal function is therefore should involve gut-protective interventions that address the overlapping and interacting effects of diarrhea, enteropathy and malnutrition to improve child survival and developmental potential in the long-term (3, 25, 35). Peptide-based enteral formulas seem to have clinical applications in malnourished children with different levels of compromised gastrointestinal function, given their association with improved gastrointestinal tolerance and absorption, better nitrogen retention/ balance, reduced diarrhea and bacterial translocation, enhanced MCT-based fat absorption and maintained/restored gut integrity as compared with free amino acid or whole-protein formulas (34, 36, 61, 63, 66, 68, 69). Future researches on epidemiology and pathogenesis addressing the factors that drive and sustain malnutrition and the causal pathway from EED to stunting are needed to develop novel approaches to improve nutritional status and linear growth of malnourished children with compromised gastrointestinal dysfunction.

## Author Contributions

AAS had primary responsibility for final content. All authors read and approved the final manuscript.

## Conflict of Interest

AAS and SE are Abbott employees. The remaining authors declare that the research was conducted in the absence of any commercial or financial relationships that could be construed as a potential conflict of interest.

## Abbreviations

EED: environmental enteric dysfunction
L/R: lactulose/rhamnose ratio
LBP: lipopolysaccharide binding protein
LCT: long-chain triglycerides
LMICs: low- and middle-income countries
MCT: medium chain triglycerides
VH: villus height
VP: villus perimeter.

## References

[1] Black RE Victora CG Walker SP Bhutta ZA Christian P de Onis M. Maternal and child undernutrition and overweight in low-income and middle-income countries. Lancet. (2013) 382:427–51. 10.1016/S0140-6736(13)60937-X 23746772

[2] Mukuku O Mutombo AM Kamona LK Lubala TK Mawaw PM Aloni MN. Predictive model for the risk of severe acute malnutrition in children. J Nutr Metab. (2019) 2019:4740825. 10.1155/2019/4740825 31354989 PMC6636463

[3] Prendergast AJ Kelly P. Interactions between intestinal pathogens, enteropathy and malnutrition in developing countries. Curr Opin Infect Dis. (2016) 29:229–36. 10.1097/QCO.0000000000000261 26967147 PMC4888918

[4] McDonald CM Olofin I Flaxman S Fawzi WW Spiegelman D Caulfield LE. The effect of multiple anthropometric deficits on child mortality: meta-analysis of individual data in 10 prospective studies from developing countries. Am J Clin Nutr. (2013) 97:896–901. 10.3945/ajcn.112.047639 23426036

[5] Bartelt LA Bolick DT Guerrant RL. Disentangling microbial mediators of malnutrition: modeling environmental enteric dysfunction. Cell Mol Gastroenterol Hepatol. (2019) 7:692–707. 10.1016/j.jcmgh.2018.12.006 30630118 PMC6477186

[6] Saunders J Smith T Stroud M. Malnutrition and undernutrition. Medicine. (2019) 47:152–8. 10.1016/j.mpmed.2010.10.007

[7] Sharkey KA Beck PL McKay DM. Neuroimmunophysiology of the gut: advances and emerging concepts focusing on the epithelium. Nat Rev Gastroenterol Hepatol. (2018) 15:765–84. 10.1038/s41575-018-0051-4 30069036

[8] Valitutti F Fasano A. Breaking down barriers: how understanding celiac disease pathogenesis informed the development of novel treatments. Dig Dis Sci. (2019) 64:1748–58. 10.1007/s10620-019-05646-y 31076989 PMC6586517

[9] Pawłowska K Umławska W Iwańczak B. A link between nutritional and growth states in pediatric patients with functional gastrointestinal disorders. J Pediatr. (2018) 199:171–7. 10.1016/j.jpeds.2018.02.069 29709346

[10] Amadi B Besa E Zyambo K Kaonga P Louis-Auguste J Chandwe K. Impaired barrier function and autoantibody generation in malnutrition enteropathy in Zambia. EBioMedicine. (2017) 22:191–9. 10.1016/j.ebiom.2017.07.017 28750860 PMC5552244

[11] Iqbal NT Syed S Sadiq K Khan MN Iqbal J Ma JZ. Study of Environmental Enteropathy and Malnutrition (SEEM) in Pakistan: protocols for biopsy based biomarker discovery and validation. BMC Pediatr. (2019) 19:247. 10.1186/s12887-019-1564-x 31331393 PMC6643315

[12] Prendergast A Kelly P. Enteropathies in the developing world; neglected effects on global health. Am J Trop Med Hyg. (2012) 86:756–63. 10.4269/ajtmh.2012.11-0743 22556071 PMC3335677

[13] Prendergast AJ Rukobo S Chasekwa B Mutasa K Ntozini R Mbuya MNN. Stunting is characterized by chronic inflammation in Zimbabwean infants. PLoS ONE. (2014) 9:e86928. 10.1371/journal.pone.0086928 24558364 PMC3928146

[14] James WPT. Effects of protein-calorie malnutrition on intestinal absorption. Ann N Y Acad Sci. (1971) 176:244–61.

[15] Bhutta ZA Berkley JA Bandsma RHJ Kerac M Trehan I Briend A. Severe childhood malnutrition. Nat Rev Dis Primers. (2017) 3:17067. 10.1038/nrdp.2017.67 28933421 PMC7004825

[16] Viteri FE Schneider RE. Gastrointestinal alterations in protein-calorie malnutrition. Med Clin North Am. (1974) 58:1487–505. 4214971 10.1016/s0025-7125(16)32085-5

[17] Patwari AK. Diarrhoea and malnutrition interaction. Indian J Pediatr. (1999) 66(Suppl. 1):S124–34. 11132459

[18] Salazar-Lindo E Allen S Brewster DR Elliott EJ Fasano A Phillips AD. Intestinal infections and environmental enteropathy: working group report of the second world congress of pediatric gastroenterology, hepatology, and nutrition. J Pediatr Gastroenterol Nutr. (2003) 39:S662–9. 10.1097/00005176-200406002-00013 15184767

[19] Brewster DR Manary MJ Menzies IS O'Loughlin EV Henry RL. Intestinal permeability in kwashiorkor. Arch Dis Child. (1997) 76:236–41. 10.1136/adc.76.3.236 9135265 PMC1717121

[20] Keusch GT Denno DM Black RE Duggan C Guerrant RL Lavery JV. Environmental enteric dysfunction: pathogenesis, diagnosis, and clinical consequences. Clin Infect Dis. (2014) 59(Suppl. 4):S207–12. 10.1093/cid/ciu485 25305288 PMC4481570

[21] Semba RD Shardell M Trehan I Moaddel R Maleta KM Ordiz MI. Metabolic alterations in children with environmental enteric dysfunction. Sci Rep. (2016) 6:28009. 10.1038/srep28009 27294788 PMC4904796

[22] Farràs M Chandwe K Mayneris-Perxachs J Amadi B Louis-Auguste J Besa E. Characterizing the metabolic phenotype of intestinal villus blunting in Zambian children with severe acute malnutrition and persistent diarrhea. PLoS ONE. (2018) 13:e0192092. 10.1371/journal.pone.0192092 29499047 PMC5834158

[23] Kvissberg MA Dalvi PS Kerac M Voskuijl W Berkley JA Priebe MG. Carbohydrate malabsorption in acutely malnourished children and infants: a systematic review. Nutr Rev. (2016) 74:48–58. 10.1093/nutrit/nuv058 26578625 PMC4684688

[24] Viteri FE Flores M Alvarado J Béhar M. Intestinal malabsorption in malnourished children before and during recovery. Relation between severity of protein deficiency and the malabsorption process. Am J Dig Dis. (1973) 18:201–11. 10.1007/BF01071974 4631820

[25] Mazahir I Rahman MA Arif MA. Studies on malabsorption in malnourished Pakistani children. Z Naturforsch C J Biosci. (1988) 43:782–6. 10.1515/znc-1988-9-1024 3149828

[26] Murphy JL Badaloo AV Chambers B Forrester TE Wootton SA Jackson AA. Maldigestion and malabsorption of dietary lipid during severe childhood malnutrition. Arch Dis Child. (2002) 87:522–5. 10.1136/adc.87.6.522 12456554 PMC1755840

[27] Schneider RE Viteri FE. Luminal events of lipid absorption in protein-calorie malnourished children; relationship with nutrition recovery and diarrhoea. II. Alterations in bile acid content of duodenal aspirates. Am J Clin Nutr. (1974) 27:788–96. 10.1093/ajcn/27.8.788 4211017

[28] Mehta HC Saini AS Singh H Dhatt PS. Biochemical aspects of malabsorption in marasmus. Br J Nutr. (1984) 51:1–6. 10.1079/bjn19840003 6418198

[29] Fitton N Thomas JS. Gastrointestinal dysfunction. Surgery. (2009) 27:492–5. 10.1016/j.mpsur.2009.09.005

[30] Denno DM Tarr PI Nataro JP. Environmental enteric dysfunction: a case definition for intervention trials. Am J Trop Med Hyg. (2017) 97:1643–6. 10.4269/ajtmh.17-0183 29016294 PMC5805039

[31] Korpe PS Petri WA Jr. Environmental enteropathy: critical implications of a poorly understood condition. Trends Mol Med. (2012) 18:328–36. 10.1016/j.molmed.2012.04.007 22633998 PMC3372657

[32] Crane RJ Jones KD Berkley JA. Environmental enteric dysfunction: an overview. Food Nutr Bull. (2015) 36(Suppl. 1):S76–87. 10.1177/15648265150361S113 PMC4472379 25902619

[33] Prendergast AJ Humphrey JH Mutasa K Majo FD Rukobo S Govha M. Assessment of environmental enteric dysfunction in the SHINE trial: methods and challenges. Clin Infect Dis. (2015) 61(Suppl. 7):S726–32. 10.1093/cid/civ848 26602300 PMC4657593

[34] Phillips VM Short N Turner C Rece J. Peptide-Based Formulas: The Nutraceuticals of Enteral Feedings. ECPN (2005). Available online at: http://www.sci-health.org/sos/html_powerpoints/Phillips_ECPN_Peptides.pdf (accessed November 11, 2019).

[35] Chen L Cekola P Telch J Cohen S Huhmann M. Pediatric nutrition needs met with a high calorie peptide-based enteral formula. J Acad Nutr Diet. (2017) 117:A30. 10.1016/j.jand.2017.06.271

[36] Brinson RR Hanumanthu SK Pitts WM. A reappraisal of the peptide-based enteral formulas: clinical applications. Nutr Clin Pract. (1989) 4:211–7. 10.1177/0115426589004006211 2513472

[37] Management of Malnutrition in Children Under Five Years. Available online at: http://motherchildnutrition.org/malnutrition-management/info/feeding-formulas-f75-f100.html (accessed January 10, 2020).

[38] What is F-75 and F-100: Twelve Differences. Available online at: https://www.publichealthnotes.com/difference-f-75-f-100/ (accessed March 23, 2020).

[39] World Health Organization. Severe Malnutrition. (2005). Available online at: https://www.who.int/nutrition/publications/severemalnutrition/9241593318_report.pdf (accessed January 10, 2020).

[40] Koretz RL Meyer JR. Elemental diets–facts and fantasies. Gastroenterology. (1980) 78:393–410. 6985599

[41] Silk DB Grimble GK Rees RG. Protein digestion and amino acid and peptide absorption. Proc Nutr Soc. (1985) 44:63–72. 10.1079/pns19850011 3885229

[42] Evelyn M Phillips ME Short NM Turner C. Peptide-Based Formulas: The Nutraceuticals of Enteral Feedings. ECPN (2005). Available online at: http://www.sci-health.org/sos/html_powerpoints/Phillips_ECPN_Peptides.pdf (accessed January 20, 2020).

[43] Shou J Ruelaz EA Redmond HP Cheng A Leon P. Kelly C. J.. Dietary protein prevents bacterial translocation from the gut. J Parenter Enteral Nutr. (1991) 15(Suppl.):29.

[44] Semba RD Trehan I Li X Moaddel R Ordiz MI Maleta KM. Environmental enteric dysfunction is associated with carnitine deficiency and altered fatty acid oxidation. EBioMedicine. (2017) 17:57–66. 10.1016/j.ebiom.2017.01.026 28122695 PMC5360565

[45] Louis-Auguste J Besa E Zyambo K Munkombwe D Banda R Banda T. Tryptophan, glutamine, leucine, and micronutrient supplementation improves environmental enteropathy in Zambian adults: a randomized controlled trial. Am J Clin Nutr. (2019) 110:1240–52. 10.1093/ajcn/nqz189 31504110 PMC6821547

[46] Vonaesch P Morien E Andrianonimiadana L Sanke H Mbecko JR Huus KE. Stunted childhood growth is associated with decompartmentalization of the gastrointestinal tract and overgrowth of oropharyngeal taxa. Proc Natl Acad Sci USA. (2018) 115:E8489–98. 10.1073/pnas.1806573115 30126990 PMC6130352

[47] Kelly P Besa E Zyambo K Louis-Auguste J Lees J Banda T. Endomicroscopic and transcriptomic analysis of impaired barrier function and malabsorption in environmental enteropathy. PLoS Negl Trop Dis. (2016) 10:e0004600. 10.1371/journal.pntd.0004600 27050312 PMC4822862

[48] Lima AA Brito LF Ribeiro HB Martins MC Lustosa AP Rocha EM. Intestinal barrier function and weight gain in malnourished children taking glutamine supplemented enteral formula. J Pediatr Gastroenterol Nutr. (2005) 40:28–35. 10.1097/00005176-200501000-00006 15625423

[49] Marc Rhoads J Wu G. Glutamine, arginine, and leucine signaling in the intestine. Amino Acids. (2009) 37:111–22. 10.1007/s00726-008-0225-4 19130170

[50] Williams EA Elia M Lunn PG. A double-blind, placebo-controlled, glutamine-supplementation trial in growth-faltering Gambian infants. Am J Clin Nutr. (2007) 86:421–7. 10.1093/ajcn/86.2.421 17684214

[51] Lima NL Soares AM Mota RM Monteiro HS Guerrant RL Lima AA. Wasting and intestinal barrier function in children taking alanyl-glutamine-supplemented enteral formula. J Pediatr Gastroenterol Nutr. (2007) 44:365–74. 10.1097/MPG.0b013e31802eecdd 17325559

[52] Costa LB Noronha FJ Roche JK Sevilleja JE Warren CA Oriá R. Novel in vitro and in vivo models and potential new therapeutics to break the vicious cycle of Cryptosporidium infection and malnutrition. J Infect Dis. (2012) 205:1464–71. 10.1093/infdis/jis216 22454464 PMC3324401

[53] Rodrigues RS Oliveira RA Li Y Zaja-Milatovic S Costa LB Braga Neto MB. Intestinal epithelial restitution after TcdB challenge and recovery from *Clostridium difficile* infection in mice with alanyl-glutamine treatment. J Infect Dis. (2013) 207:1505–15. 10.1093/infdis/jit041 23359592 PMC3627196

[54] Fischer DD Kandasamy S Paim FC Langel SN Alhamo MA Shao L. Protein malnutrition alters tryptophan and angiotensin-converting enzyme 2 homeostasis and adaptive immune responses in human rotavirus-infected gnotobiotic pigs with human infant fecal microbiota transplant. Clin Vaccine Immunol. (2017) 24:e00172–17. 10.1128/CVI.00172-17 28637803 PMC5583476

[55] Divanovic S Sawtell NM Trompette A Warning JI Dias A Cooper AM. Opposing biological functions of tryptophan catabolizing enzymes during intracellular infection. J Infect Dis. (2012) 205:152–61. 10.1093/infdis/jir621 21990421 PMC3242739

[56] Blanton LV Charbonneau MR Salih T Barratt MJ Venkatesh S Ilkaveya O. Gut bacteria that prevent growth impairments transmitted by microbiota from malnourished children. Science. (2016) 351:aad3311. 10.1126/science.aad3311 26912898 PMC4787260

[57] Smith JL Arteaga C Heymsfield SB. Increased unreagenesis and impaired nitrogen use during infusion of a synthetic amino acid formula. N Engl J Med. (1982) 306:1013–8. 10.1056/NEJM198204293061702 6801516

[58] Matthews DM Adibi SA. Peptide absorption. Gastroenterology. (1976) 71:151–61. 10.1016/S0016-5085(76)80117-5 776732

[59] Brinson RR Kolts BE. Hypoalbuminemia as an indicator of diarrheal incidence in critically ill patients. Crit Care Med. (1987) 15:506–9. 10.1097/00003246-198705000-00011 3105959

[60] Liu MY Tang HC Hu SH Chang SJ. Peptide-based enteral formula improves tolerance and clinical outcomes in abdominal surgery patients relative to a whole protein enteral formula. World J Gastrointest Surg. (2016) 8:700–5. 10.4240/wjgs.v8.i10.700 27830042 PMC5081552

[61] Alexander DD Bylsma LC Elkayam L Nguyen DL. Nutritional and health benefits of semi-elemental diets: a comprehensive summary of the literature. World J Gastrointest Pharmacol Ther. (2016) 7:306–19. 10.4292/wjgpt.v7.i2.306 27158547 PMC4848254

[62] DeLegge MH. Enteral feeding. Curr Opin Gastroenterol. (2008) 24:184–9. 10.1097/MOG.0b013e3282f4dbab 18301269

[63] Ibrahim H Mansour M El Gendy YG. Peptide-based formula versus standard-based polymeric formula for critically ill children: is it superior for patients' tolerance? Arch Med Sci. (2020) 16:592–6. 10.5114/aoms.2020.94157 32399107 PMC7212209

[64] Holt PR. Medium chain triglycerides: a useful adjunct in nutritional therapy. Gastroenterology. (1967) 53:961–6. 4863725

[65] Smart KM Alex G Hardikar W. Feeding the child with liver disease: a review and practical clinical guide. J Gastroenterol Hepatol. (2011) 26:810–5. 10.1111/j.1440-1746.2011.06687.x 21299619

[66] Bach AC Babayan VK. Medium-chain triglycerides: an update. Am J Clin Nutr. (1982) 36:950–62. 10.1093/ajcn/36.5.950 6814231

[67] Sulkers EJ von Goudoever JB Leunisse C Wattimena JL Sauer PJ. Comparison of two preterm formulas with or without addition of medium-chain triglycerides (MCTs). I: Effects on nitrogen and fat balance and body composition changes. J Pediatr Gastroenterol Nutr. (1992) 15:34–41. 1403448 10.1097/00005176-199207000-00006

[68] Tantibhedhyangkul P Hashim SA. Medium-chain triglyceride feeding in premature infants: effects on calcium and magnesium absorption. Pediatrics. (1978) 61:537–45. 662478

[69] Graham GG Baertl JM Cordano A Morales E. Lactose-free, medium-chain triglyceride formulas in severe malnutrition. Am J Dis Child. (1973) 126:330–5. 4800365 10.1001/archpedi.1973.02110190292008

[70] Meredith JW Ditesheim JA Zaloga GP. Visceral protein levels in trauma patients are greater with peptide diet than intact protein diet. J Trauma. (1990) 30:825–8. 2116533 10.1097/00005373-199007000-00011

[71] Zaloga GP. Studies comparing intact protein, peptide, and amino acid formulas. In: Bounous G, editor. Elemental Diets in Clinical Situations. Boca Raton, FL: CRC Press (1993). p. 201–17.

[72] Tickell KD Atlas HE Walson JL. Environmental enteric dysfunction: a review of potential mechanisms, consequences and management strategies. BMC Med. (2019) 17:181. 10.1186/s12916-019-1417-3 31760941 PMC6876067

[73] Stephenson KB Agapova SE Divala O Kaimila Y Maleta KM Thakwalakwa C. Complementary feeding with cowpea reduces growth faltering in rural Malawian infants: a blind, randomized controlled clinical trial. Am J Clin Nutr. (2017) 106:15007. 10.3945/ajcn.117.160986 29092882 PMC6482976

[74] Agapova SE Stephenson KB Divala O Kaimila Y Maleta KM Thakwalakwa C. Additional common bean in the diet of Malawian children does not affect linear growth, but reduces intestinal permeability. J Nutr. (2018) 148:267–74. 10.1093/jn/nxx013 29490090

[75] Cheng WD Wold KJ Bollinger LB Ordiz MI Shulman RJ Maleta KM. Supplementation with lactoferrin and lysozyme ameliorates environmental enteric dysfunction: a double-blind, randomized, placebo-controlled trial. Am J Gastroenterol. (2019) 114:671–8. 10.14309/ajg.0000000000000170 30829679

[76] De-Regil LM Suchdev PS Vist GE Walleser S Peña-Rosas JP. Home fortification of foods with multiple micronutrient powders for health and nutrition in children under two years of age. Cochrane Database Syst Rev. (2011) 9:CD008959. 10.1002/14651858.CD008959.pub2 21901727

[77] Bierut T Duckworth L Grabowsky M Ordiz MI Laury ML Callaghan-Gillespie M. The effect of bovine colostrum/egg supplementation compared with corn/soy flour in young Malawian children: a randomized, controlled clinical trial. Am J Clin Nutr. (2020) 113:nqaa325. 10.1093/ajcn/nqaa325 33330913

[78] Parpia TC Elwood SE Scharf RJ McDermid JM Wanjuhi AW Rogawski McQuade ET. Baseline characteristics of study participants in the early life interventions for childhood growth and development in Tanzania (ELICIT) trial. Am J Trop Med Hyg. (2020) 103:1397–404. 10.4269/ajtmh.19-0918 32783799 PMC7543831

[79] Hearing SD. Refeeding syndrome. BMJ. (2004) 328:908–9. 10.1136/bmj.328.7445.908 15087326 PMC390152

[80] Ponzo V Pellegrini M Cioffi I Scaglione L Bo S. The Refeeding Syndrome: a neglected but potentially serious condition for inpatients. A narrative review. Intern Emerg Med. (2021) 16:49–60. 10.1007/s11739-020-02525-7 33074463 PMC7843537

[81] Day AS. The impact of exclusive enteral nutrition on the intestinal microbiota in inflammatory bowel disease. AIMS Microbiol. (2018) 4:584–93. 10.3934/microbiol.2018.4.584 31294235 PMC6613331

[82] Gerasimidis K Bertz M Hanske L Junick J Biskou O Aguilera M. Decline in presumptively protective gut bacterial species and metabolites are paradoxically associated with disease improvement in pediatric Crohn's disease during enteral nutrition. Inflamm Bowel Dis. (2014) 20:861–71. 10.1097/MIB.0000000000000023 24651582

[83] Quince C Ijaz UZ Loman N Eren AM Saulnier D Russell J. Extensive modulation of the fecal metagenome in children with Crohn's disease during exclusive enteral nutrition. Am J Gastroenterol. (2015) 110:1718–29. 10.1038/ajg.2015.357 26526081 PMC4697132

[84] Hansen T Duerksen DR. Enteral nutrition in the management of pediatric and adult Crohn's disease. Nutrients. (2018) 10:537. 10.3390/nu10050537 29701656 PMC5986417

[85] Dunn KA Moore-Connors J MacIntyre B Stadnyk AW Thomas NA Noble A. Early changes in microbial community structure are associated with sustained remission after nutritional treatment of pediatric Crohn's disease. Inflamm Bowel Dis. (2016) 22:2853–62. 10.1097/MIB.0000000000000956 27805918

[86] Krezalek MA Yeh A Alverdy JC Morowitz M. Influence of nutrition therapy on the intestinal microbiome. Curr Opin Clin Nutr Metab Care. (2017) 20:131–7. 10.1097/MCO.0000000000000348 27997410

[87] Chassaing B Koren O Goodrich JK Poole AC Srinivasan S Ley RE. Dietary emulsifiers impact the mouse gut microbiota promoting colitis and metabolic syndrome. Nature. (2015) 519:92–6. 10.1038/nature14232 25731162 PMC4910713

[88] Grier A Qiu X Bandyopadhyay S Holden-Wiltse J Kessler HA Gill AL. Impact of prematurity and nutrition on the developing gut microbiome and preterm infant growth. Microbiome. (2017) 5:158. 10.1186/s40168-017-0377-0 29228972 PMC5725645

[89] Diederen K Li JV Donachie GE de Meij TG de Waart DR Hakvoort TBM. Exclusive enteral nutrition mediates gut microbial and metabolic changes that are associated with remission in children with Crohn's disease. Sci Rep. (2020) 10:18879. 10.1038/s41598-020-75306-z 33144591 PMC7609694

